# Quantifying tissue optical properties of human heads *in vivo* using continuous-wave near-infrared spectroscopy and subject-specific three-dimensional Monte Carlo models

**DOI:** 10.1117/1.JBO.27.8.083021

**Published:** 2022-06-22

**Authors:** Tzu-Chia Kao, Kung-Bin Sung

**Affiliations:** a National Taiwan University, Graduate Institute of Biomedical Electronics and Bioinformatics, Taipei, Taiwan; b National Taiwan University, Department of Electrical Engineering, Taipei, Taiwan; c National Taiwan University, Molecular Imaging Center, Taipei, Taiwan

**Keywords:** optical properties, near-infrared spectroscopy, tissues, Monte Carlo method

## Abstract

**Significance:**

Quantifying subject-specific optical properties (OPs) including absorption and transport scattering coefficients of tissues in the human head could improve the modeling of photon propagation for the analysis of functional near-infrared spectroscopy (fNIRS) data and dosage quantification in therapeutic applications. Current methods employ diffuse approximation, which excludes a low-scattering cerebrospinal fluid compartment and causes errors.

**Aim:**

This work aims to quantify OPs of the scalp, skull, and gray matter *in vivo* based on accurate Monte Carlo (MC) modeling.

**Approach:**

Iterative curve fitting was applied to quantify tissue OPs from multidistance continuous-wave NIR reflectance spectra. An artificial neural network (ANN) was trained using MC-simulated reflectance values based on subject-specific voxel-based tissue models to replace MC simulations as the forward model in curve fitting. To efficiently generate sufficient data for training the ANN, the efficiency of MC simulations was greatly improved by white MC simulations, increasing the detectors’ acceptance angle, and building a lookup table for interpolation.

**Results:**

The trained ANN was six orders of magnitude faster than the original MC simulations. OPs of the three tissue compartments were quantified from NIR reflectance spectra measured at the forehead of five healthy subjects and their uncertainties were estimated.

**Conclusions:**

This work demonstrated an MC-based iterative curve fitting method to quantify subject-specific tissue OPs *in-vivo*, with all OPs except for scattering coefficients of scalp within the ranges reported in the literature, which could aid the modeling of photon propagation in human heads.

## 1 Introduction

Human brain activities have long been of great interest to many fields stretching from basic neuroscience, branches of medicine, and education to even brain-computer interface. Functional magnetic resonance imaging (fMRI) is the gold standard method of brain activity monitoring with great three-dimensional (3D) spatial resolution over the whole brain. However, the temporal resolution of fMRI is low, and the subject or patient is restricted to the supine position in an MRI scanner, which is expensive and not readily accessible. Functional near-infrared spectroscopy (fNIRS) employs low-cost and compact instruments to monitor brain activities with moderate temporal and spatial resolution. Since fNIRS measurements can be taken while the subject or patient is in any position or even moving, their applications are more versatile than fMRI.

In fNIRS, red to near-infrared light in at least two wavelengths is shone on the head and intensity changes of the diffuse reflection light are measured. The cerebral cortex consumes oxygen during neuronal activation, which induces increases in local blood volume and blood flow. The concentrations of oxyhemoglobin (HbO) and deoxyhemoglobin (Hb) in the active area change consequently. Quantification of hemoglobin concentration changes (ΔC) is achieved based on the modified Beer–Lambert law (MBLL)^1^

$$\Delta \mathrm{OD}=\mathrm{PL}\times (\varepsilon_{\mathrm{Hb}}\times \Delta C_{\mathrm{Hb}}+\varepsilon_{\mathrm{HbO}}\times \Delta C_{\mathrm{HbO}}),$$ (1)

where ΔOD is the logarithm of the ratio of diffuse reflectance intensities between two-time points, PL is the light pathlength in the cerebral cortex, and ε is the molar extinction coefficients of the hemoglobins. In essence, the concentration changes can be calculated from intensity measurements if the light pathlength in the corresponding tissue compartment can be quantified. Many fNIRS studies have used a pathlength calculated using a fixed differential pathlength factor,^2^ which is the ratio between the pathlength in tissue and the distance between the source and detector at the scalp surface. Subject-specific pathlengths can be obtained based on a homogeneous tissue model and optical properties (OPs) including the absorption coefficient ($\mu_{a}$) and the transport scattering coefficient ($\mu_{s}^{\prime }$)^3^ measured by for example time-resolved (TR)^4^ or frequency-domain (FD)^5^ reflectance spectroscopy.

Although MBLL is widely adopted to achieve acceptable results for qualitatively assessing the general trend in hemodynamic changes, its ability to accurately quantify the concentration changes is debatable because the cerebral cortex, the target region of fNIRS, only accounts for a small portion of light attenuation in the head, which is known as the partial volume effect.^6^^–^^8^ To separate the cerebral contribution from the typically stronger influence of the extra-cerebral tissue on the measured intensity changes, it is important to separately estimate partial pathlengths in the brain and the extra-cerebral tissue compartment,^9^^,^^10^ which can be achieved by Monte Carlo (MC) simulations of light propagations in a realistic head model such as that segmented from MRI scans of the head.^11^ However, OPs of major tissue types in the head measured from *ex-vivo* tissue samples show wide variations among studies,^12^ which can be attributed to different sample preparation procedures^13^ and intersubject variability. Moreover, there were substantial intersubject variations in pathlengths estimated by finite-element calculations of light propagation using the same OPs on 40 subject-specific head models.^14^ Therefore, it is imperative to develop a noninvasive method for quantifying tissue OPs in the head of individual subjects to account for intersubject variabilities.

Precisely measuring head tissue OPs for individual subjects can also help other applications. For example, photobiomodulation is a technique that uses red or near-infrared light to stimulate or even heal tissue and has been applied to the human brain against dementia, Alzheimer’s disease, and Parkinson’s disease. The beneficial effect of photobiomodulation shows the biphasic dose response,^15^ which requires the dosage to be within a proper range. Research has been done to simulate the fraction of energy delivered to the human brain under transcranial stimulation,^12^ and found that the dosage in the brain is greatly affected by the head tissue OPs.

NIRS is often used to noninvasively measure the head OPs. The TR and FD NIRS systems have been used to measure head OPs in many studies,^16^^–^^18^ but the instruments are more complicated and expensive than continuous-wave (CW) NIRS systems. CW NIRS systems have also been used to quantify OPs of piglets’ brains^19^ and human brain.^20^ In these studies, the diffusion approximation of the radiative transfer equation for a semi-infinite homogeneous model^19^ or a two-layered (i.e., extra-cerebral and cerebral) model^16^^–^^18^^,^^20^ was used to simulate diffuse reflectance spectra. A low-scattering cerebrospinal fluid (CSF) layer was not included in the models due to the limitation of diffusion approximation to highly scattering media. Indeed, using the diffusion approximation solution to simulate light propagation in a realistic head model containing a low-scattering CSF compartment could lead to significant errors.^21^ However, excluding the low-scattering CSF layer could also significantly affect the simulation results of photon propagation in the head.^22^^,^^23^ The MC method, on the other hand, is considered the gold standard and has been implemented to simulate light propagation in realistic head models.^24^ It has not been used in iteratively curve fitting of NIRS data for quantifying OPs in the human head due to its relatively long time to simulate photon propagation in a deep, complex model like the human head.

This study aimed to establish a practical procedure to estimate the OPs of cerebral and extracerebral tissue compartments *in-vivo* using MC-based iterative curve fitting of CW NIRS data. Realistic human head models consisting of six tissue compartments were constructed from MRI scans of each subject. To overcome the challenge of huge time cost in running MC simulations, we trained an artificial neural network (ANN) to replace MC simulations as the forward model that calculated diffuse reflectance from given OPs.^25^ However, due to the number and ranges of OPs required for this study a very large number of MC-simulated reflectance was needed to train the ANN. Therefore, the efficiency of generating the training data was enhanced by several techniques to prevent prohibitively long simulation time. First, white MC (WMC) was used to quickly calculate the reflectance for various $\mu_{a}$ from only one simulation result obtained using each combination of $\mu_{s}^{\prime }$. Second, WMC simulations were run for detectors with a high acceptance angle to increase their efficiency, and a linear regression model was built to convert the simulated reflectance to that corresponding to the actual acceptance angle of the detectors used to collect *in-vivo* NIRS data. Finally, the number of $\mu_{s}^{\prime }$ combinations in the training data was increased by spline interpolation of simulated reflectance obtained from 2808 $\mu_{s}^{\prime }$ combinations covering the reported ranges.^12^ The trained ANN was six orders of magnitude faster than the MC simulation, making iterative curve fitting practical.

The fitting process was tested on simulated target spectra without noise and with proper noise to analyze the theoretical performance of the proposed method. *In-vivo* reflectance spectra were measured from five healthy volunteers with a broadband CW NIRS system built in-house, and $\mu_{s}^{\prime }$ and $\mu_{a}$ of the scalp, skull, and gray matter were quantified. Thanks to the highly efficient ANN forward model, uncertainty in the extracted OPs due to fluctuations in the measured *in-vivo* spectra were estimated.

## 2 Methods

**Fig. 1:**
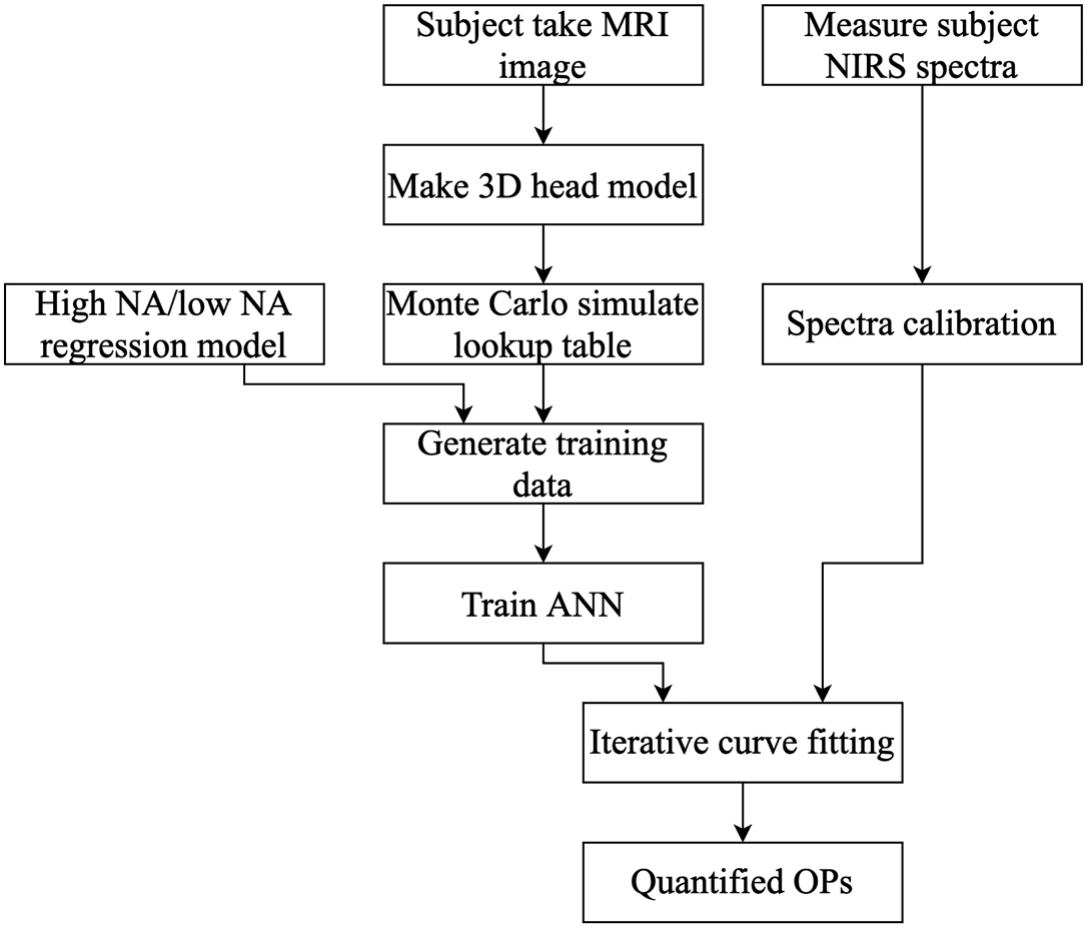
Flow chart of building an ANN as a forward model and quantifying tissue OPs from measurements.

A flow chart of the proposed method is shown in Fig. 1. T1-weighted head MRI scan was performed on nine subjects to obtain the anatomical structure of the head and build a 3D model for each subject. One ANN forward model was trained for each subject and used in iterative curve fitting to quantity tissue OPs of the scalp, skull, and gray matter. Due to changes in the NIRS system, only five of the nine subjects underwent *in-vivo* CW NIRS measurements on the right forehead and their tissue OPs were quantified using the proposed method. The human study was approved by the institutional review board of National Taiwan University, and all participants provided written informed consent.

### 2.1 Head Model Preparation

**Fig. 2:**
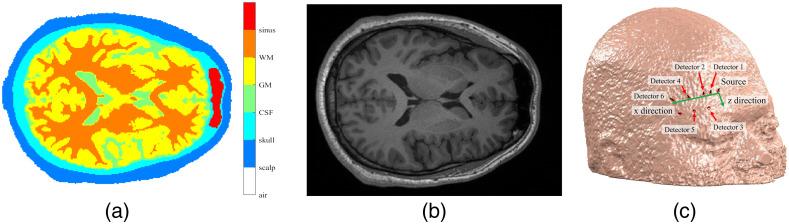
The head model (a) a slice of the segmented head model, (b) the corresponding MRI scan, and (c) positions of the source and detector fibers at the head surface.

To make the simulation closer to reality, we performed T1-weighted MRI head scans on nine subjects to get anatomical structure specific to each subject. The voxel size was $0.93\times 0.93\times 0.93\;{\mathrm{mm}}^{3}$. The MRI data were segmented using SPM^26^ into five compartments including scalp, skull, CSF, gray matter (GM), and white matter (WM). The region with relatively low MRI signal intensity in the front head was considered as sinus and manually segmented to be the sixth compartment. One slice of the segmented head model and its corresponding MRI scan slice are shown in Figs. 2(a) and 2(b), respectively. The surface of the scalp was transformed into a mesh and the Mesh2EEG^27^ function was performed to find locations of the EEG 10-5 system^28^ on the surface. To measure reflectance spectra from the right forehead the source was placed at the EEG Fp2 position and the locations of the source and six detectors are shown in Fig. 2(c). The source-to-detector separation (SDS) for the six detectors was 0.8, 1.5, 2.12, 3, 3.35, and 4.5 cm, respectively.

OPs needed by the MC method to simulate photon energy propagation in tissue include absorption coefficient ($\mu_{a}$), scattering coefficient ($\mu_{s}$), refractive index (n), and anisotropic factor (g). Ranges of the OPs for each tissue type are listed in Table 1. The refractive index was assumed constant throughout the head to simplify MC simulations. The refractive index of the medium outside the head was set to 1.457, which equaled to that of detector fibers to simulate the exit angle of photons properly. The space between the source and detector fibers in the NIRS system was a soft pad with n around 1.42, which was close to the value used in the simulations.

**Table 1:** Ranges of OPs for each compartment or medium.

Tissue type	$\mu_{a}$ (1/cm)	$\mu_{s}^{\prime }$ (1/cm)	n	g
Outer medium	0	0	1.457	—
Scalp	0.1 to 0.6	5 to 35	1.4	0.9
Skull	0.05 to 0.45	5 to 35	1.4	0.9
CSF	0.015 to 0.1	1 to 3.7	1.4	0.9
GM	0.05 to 0.5	5 to 35	1.4	0.9
WM	0.025 to 0.25	15 to 105	1.4	0.9
Sinus	0	0	1	—

In the diffuse regime, effects of $\mu_{s}$ and g on photon propagation in tissue can be combined into the transport scattering coefficient ($\mu_{s}^{\prime }$), which is expressed as

$$\mu_{s}^{\prime }=\mu_{s}\times (1-g).$$ (2)

The anisotropy factor of tissue was assumed to be constant since its effect was considered by $\mu_{s}^{\prime }$. Wavelength-dependent $\mu_{s}^{\prime }$ were assumed to follow an inverse power-law function of wavelength λ^29^ and calculated with coefficients A and K as

$$\mu_{s}^{\prime }(\lambda )=A\times \lambda^{-k}.$$ (3)

**Fig. 3:**
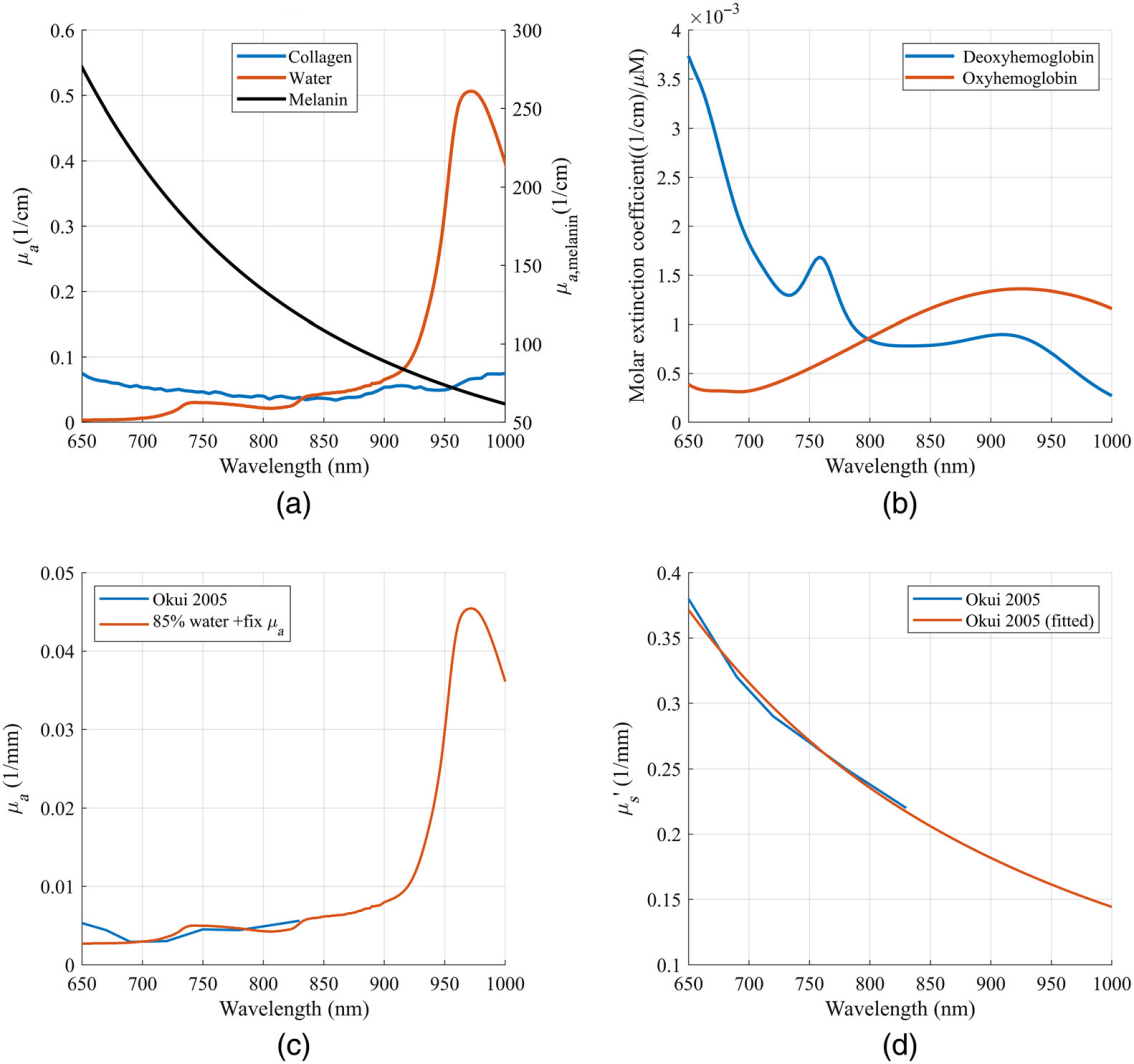
(a) Absorbance spectra of 100% (v/v) collagen,^30^ water,^31^ and melanin^32^; (b) molar extinction coefficient spectra of oxy- and deoxy-hemoglobins^33^; (c) the absorption coefficient spectrum of CSF^34^; (d) the transport scattering coefficient spectrum of CSF.^34^

The wavelength dependence of tissue $\mu_{a}$ was assumed to be known based on absorption coefficients of major tissue chromophores in the wavelength range analyzed in this study. Exploiting the wavelength dependences of $\mu_{s}^{\prime }$ and $\mu_{a}$ could improve the efficiency and robustness of the inverse model applied on broadband spectra since only a few unknown parameters need to be determined instead of two OPs for each wavelength. The $\mu_{a}(\lambda )$ of a tissue compartment was calculated as

$$\mu_{a,\text{tissue}}(\lambda )=2.303\times [\varepsilon_{\mathrm{HbO}}(\lambda )\times {\mathrm{StO}}_{2}+\varepsilon_{\mathrm{Hb}}(\lambda )\times (1-{\mathrm{StO}}_{2})]\times \mathrm{tHB}+\overset{m}{\underset{i=1}{\sum }}\mu_{a}^{i}(\lambda )\times C_{i},$$ (4)

where $\varepsilon_{\mathrm{Hb}}$ and $\varepsilon_{\mathrm{HbO}}$ are the molar extinction coefficient of Hb and HbO, respectively, ${\mathrm{StO}}_{2}$ is the tissue oxygen saturation, tHB is the total hemoglobin concentration, $\mu_{a}^{i}$ is the absorption coefficient of 100% (v/v) of the i’th absorber other than hemoglobin, and $C_{i}$ is the volume fraction of the absorber. The ε and $\mu_{a}^{i}$ spectra used in this study are shown in Figs. 3(a) and 3(b).

$\mu_{a}$ and $\mu_{s}^{\prime }$ spectra of the CSF were assumed to have fixed values adopted from the literature as shown in Figs. 3(c) and 3(d) since they are relatively small and barely affect the reflectance spectra. The $\mu_{a}$ of WM was set to be half of the $\mu_{a}$ of GM, and the $\mu_{s}^{\prime }$ of WM was set to be 3 times the $\mu_{s}^{\prime }$ of GM.^35^ Since WM depths in the segmented models are between 16 and 20 mm, the approximation of WM OPs was not expected to influence the results of quantified tissue OPs. The tissue parameters used to calculate $\mu_{s}^{\prime }(\lambda )$ and $\mu_{a}(\lambda )$ of the five tissue compartments are summarized in Table 2.

**Table 2:** Tissue parameters used to calculate OPs of the five major tissue compartments.

Tissue	Variable tissue parameters	Fixed tissue parameters
Scalp	tHB, ${\mathrm{StO}}_{2}$, $C_{\text{melanin}}$, A, K	$C_{\text{water}}=75\%$, $C_{\text{collagen}}=2\%$
Skull	tHB, $StO_{2}$, A, K	
CSF	None	Literature reported values
GM	tHB, ${\mathrm{StO}}_{2}$, A, K	$C_{\text{water}}=75\%$
WM	None	$\mu_{a,\mathrm{WM}}=\frac{\mu_{a,\mathrm{GM}}}{2}$, $\mu_{s,\mathrm{WM}}^{\prime }=3\times \mu_{S,\mathrm{GM}}^{\prime }$

### 2.2 Monte Carlo Simulation and Acceleration

An open-source graphics-processing units (GPU) accelerated MC simulation tool, Monte Carlo eXtreme (MCX),^36^ was used to perform simulations of photon propagation in voxel-based head models. To accelerate the simulations, we performed WMC in which $\mu_{a}$ of all tissue types were set to zero and the pathlength of each detected photon in each tissue compartment was recorded. After the simulation with one set of $\mu_{s}^{\prime }$ was done, the reflectance of tissue models with the same set of $\mu_{s}^{\prime }$ and any $\mu_{a}$ combination could be quickly calculated by summing the attenuated weight of every detected photon according to microscopic BLL^37^

$$R=[\overset{N}{\underset{i=1}{\sum }}e^{-\overset{5}{\underset{l=1}{\sum }}\mu_{a,l}\times \mathrm{PL}(i,l)}]/N_{\text{total}},$$ (5)

where R is the calculated reflectance, N is the number of detected photons, $\mu_{a,l}$ is the absorption coefficient of tissue compartment l, PL(i,l) is the pathlength of the i’th photon in tissue compartment l from the simulation, and $N_{\text{total}}$ is the number of launched photons. WMC simulations were performed using 2808 $\mu_{s}^{\prime }$ combinations whose values for each of the four tissue compartments are listed in Table 3. The number of sampled $\mu_{s}^{\prime }$ values for each tissue compartment was determined empirically and roughly followed the sensitivity of the reflectance to each compartment’s $\mu_{s}^{\prime }$. Results of WMC simulations, i.e., N and PL(i,l) of detected photons, were recorded in a lookup table, which contained 2808 entries corresponding to the simulated $\mu_{s}^{\prime }$ combinations.

**Table 3:** $\mu_{s}^{\prime }$ of each tissue compartment used to construct the lookup table.

Tissue	$\mu_{s}^{\prime }$ (1/cm)	Number of points
Scalp	5, 7.5, 10, 12.5, 15, 17.5, 20, 22.5, 25, 27.5, 30, 32.5, 35	13
Skull	5, 8.75, 12.5, 16.25, 20, 23.75, 27.5, 31.25, 35	9
CSF	1, 1.9, 2.8, 3.7	4
GM	5, 11, 17, 23, 29, 35	6

To further reduce the number of launched photons and hence the time needed by MC simulations while keeping the results converged, we set the numerical aperture (NA) of detector fibers in MC simulations to be 1.0, which collected significantly more photons than the NA=0.12 detector fibers actually used in the CW NIRS system described in Sec. 2.3. An NA-conversion linear regression model was developed to convert simulation results of reflectance collected by NA=1.0 fibers into those collected by NA=0.12 fibers. The resultant NA-conversion model took six inputs including logarithm of the reflectance, logarithm of the number of detected photons, $\mu_{a,\text{scalp}}$, $\mu_{a,\text{skull}}$, $\mu_{s,\text{scalp}}^{\prime }$, and $\mu_{s,\text{skull}}^{\prime }$. The output of the NA-conversion model was the ratio between the reflectance collected by NA=0.12 fibers and that by NA=1.0 fibers. Each data point in the lookup table was simulated using MCX with $10^{9}$ photons and a detector NA=1.0.

After MC simulations were done, we used the lookup table to generate data for training ANN forward models. We randomly chose 3000 $\mu_{a}$ combinations within the ranges reported in Table 1 and calculated the corresponding reflectance using Eq. (5) for each $\mu_{s}^{\prime }$ combination in the table. Then the reflectance simulated by NA=1.0 fibers was converted into reflectance detected by NA=0.12 fibers using the NA-conversion model. Since the error introduced by interpolation was rather small under these $\mu_{s}^{\prime }$ sampling intervals, we could augment the amount of training data by interpolation between the simulated $\mu_{s}^{\prime }$ values. Based on the 2808 $\mu_{s}^{\prime }$ combinations in the lookup table, more reflectance values for other $\mu_{s}^{\prime }$ combinations could be readily obtained through spline interpolation. We randomly chose 3000 $\mu_{s}^{\prime }$ combinations and calculated their reflectance values, producing a total of (2808+3000)×3000=17.4 million OP combinations with reflectance values for training ANN forward models in a reasonable time.

The 17.4 million sets of OP/reflectance data for each subject were split into training, validation, and test datasets in fractions of 75%, 10%, and 15% respectively. Inputs to the ANN models were $\mu_{a}$ and $\mu_{s}$ of the four major tissue compartments in Table 3, and outputs were reflectance values received by the six detectors. We used a fully connected neural network with four hidden layers consisting of 850, 550, 300, and 150 neurons, respectively.

### 2.3 Broadband Near-Infrared Spectroscopy Instrument

**Fig. 4:**
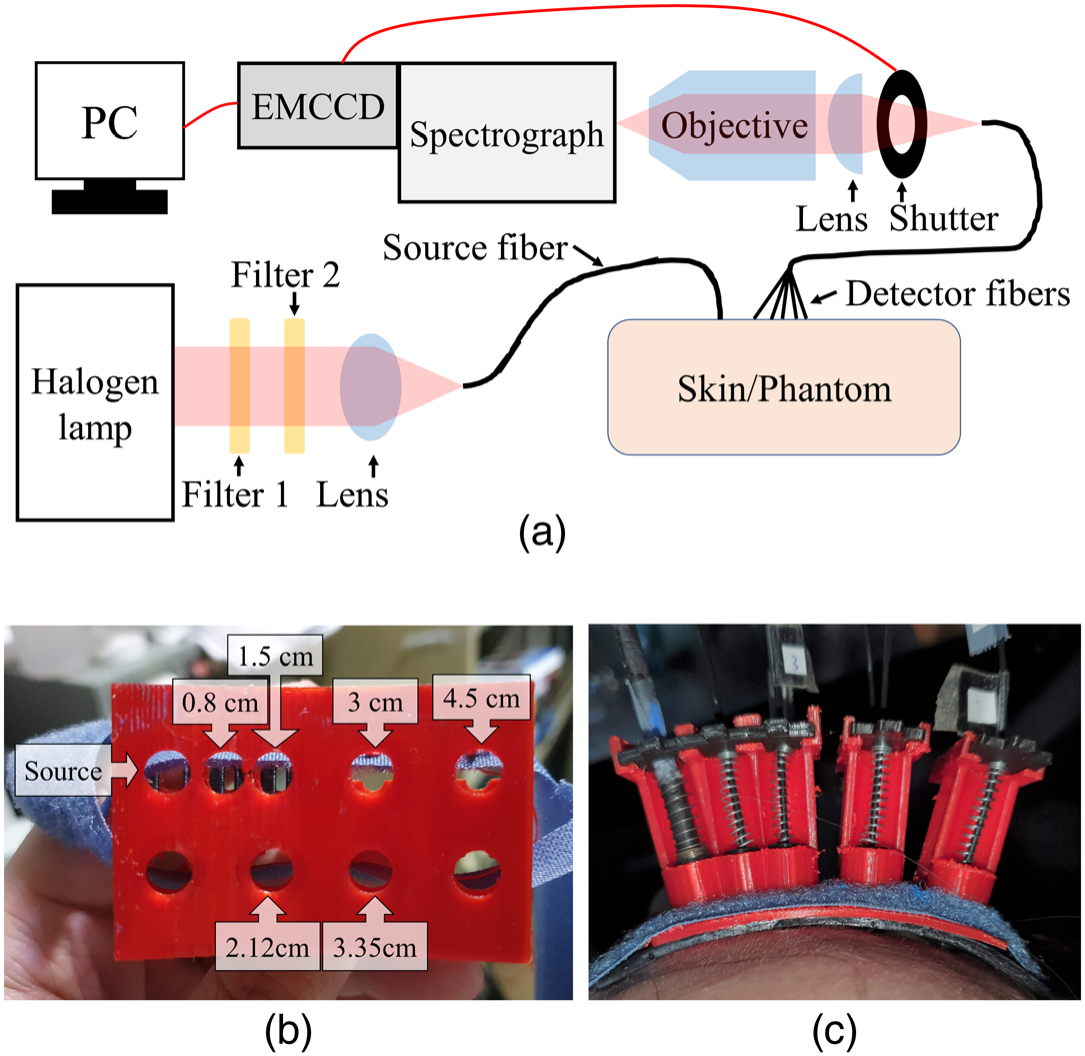
(a) Schematic diagram of the CW NIRS system, and photographs of (b) the bottom view of the holder showing locations of the fibers, and (c) the holder installed on the forehead.

We built a broadband CW NIRS system to measure *in-vivo* diffuse reflectance spectra at six SDSs from the forehead of five healthy volunteers. A schematic diagram of the system is shown in Fig. 4(a). We used a quartz tungsten halogen lamp (66997-250Q-R085, Newport Corporation, Irvine, California, United States) as the light source. Two filters were used to filter out wavelengths below 650 nm or above 1050 nm, and the remaining light was guided to the subjects by a bundle of 16 fibers whose NA was 0.39. The outer diameter of the active area of the source bundle was about 4.2 mm. The light remitted by tissue was collected by six detector fibers with a core diameter of 0.4 mm, NA of 0.12, and SDS of 0.8, 1.5, 2.12, 3, 3.35, and 4.5 cm, respectively. For convenient reference in the manuscript, the detectors were numbered as 1 to 6, respectively. A lens and an objective lens were used to image the end-face of the detector fibers to the entrance slit of an imaging spectrograph (HOLOSPEC-F/1.8- NIR, Oxford Instruments, Abingdon, Oxfordshire, United Kingdom), and spectra were measured by an electron-multiplying charge-coupled device (EMCCD) (Newton DU970P, Oxford Instruments) with a spectral resolution of about 4 nm. To make sure that the contact between the fibers and scalp was stable, we 3D-printed a curved holder and used springs as shown in Figs. 4(b) and 4(c) to press the fibers against the scalp with consistent pressure. The holder interfaced the scalp surface with a soft black pad made of polydimethylsiloxane (PDMS) to ensure stable and tight contact with the scalp surface. Ultrasound gel was smeared on the scalp near the measured site before the holder was mounted to prevent any air gap between the fibers and the scalp. The holder was placed on the subjects’ right forehead with the source fiber bundle pointing to the EEG Fp2 position.

We used the NIRS system to measure reflectance spectra from five healthy male subjects aging between 24 and 47. We repeated the procedure of applying the holder/fibers and acquiring spectra for at least three times on each subject to make sure that the contact between the fibers and scalp was stable and to estimate the variations in measured reflectance intensities. Coefficients of variation (CVs) of the repeatedly measured reflectance intensities at each SDS were averaged over all wavelengths and subjects, and results are listed in Table 4.

**Table 4:** Coefficients of variation (CV) of repeated *in-vivo* reflectance spectra measurements on each subject. The numbers shown are the average over all wavelengths and subjects.

SDS (cm)	0.8	1.5	2.12	3	3.35	4.5
CV (%)	3.0	4.2	5.1	5.2	5.4	12.1

### 2.4 Iterative Curve Fitting

Measured *in-vivo* reflectance spectra were calibrated using six homogeneous phantoms made of PDMS, ${\mathrm{TiO}}_{2}$ and India ink. After $\mu_{s}^{\prime }(\lambda )$ and $\mu_{a}(\lambda )$ of the phantoms were measured by an integrating sphere and the inverse adding-doubling method, we simulated reflectance spectra of the phantoms with CUDAMCML^38^ and performed calibrating linear regression^39^ of the simulated reflectance against the measured reflectance of the phantoms. The resultant calibrating regression equation was applied to calibrate *in-vivo* reflectance spectra to the absolute reflectance, which were compared with MC simulated reflectance. The average of multiple measured spectra of the same subject after calibration was taken as the target spectra.

We used iterative curve fitting to find values for the variable tissue parameters listed in Table 2 that minimized the root-mean-square (RMS) percent error between simulated spectra and calibrated *in-vivo* or simulated test spectra. The spectral error was calculated as

$$\text{Spectral error}=\sqrt{\frac{\overset{n_{d}}{\underset{d=1}{\sum }}\overset{n_{\lambda }}{\underset{i=1}{\sum }}{\left(\frac{R_{s}(d,i)}{R_{m}(d,i)}-1\right)}^{2}}{n_{d}\times n_{\lambda }}},$$ (6)

where $n_{d}$ is the number of detectors, $n_{\lambda }$ is the number of wavelengths considered, $R_{s}$ is the simulated reflectance in the current iteration, and $R_{m}$ is the measured reflectance after calibration or simulated reflectance of the test spectra. We chose 22 wavelengths nonevenly distributed between 700 and 880 nm to perform the fitting. Initial values of the tissue parameters to be determined were chosen from a pool of 5000 sets of randomly selected parameters. Each set of measured spectra to be fitted was first compared with presimulated spectra corresponding to the 5000 sets of parameters, from which 20 sets of parameters resulting in the smallest spectral error to the target spectra were chosen as initial values. The target spectra were fitted 20 times to address the local minimum problem. In each iteration, the temporarily guessed tissue parameters were converted to OPs, which in turn were sent to the ANN model to get simulated spectra. The iterative curve fitting function “fmincon” with a sequential quadratic programming algorithm provided by MATLAB^®^ (MathWorks, Inc., Natick, Massachusetts, United States) was used to minimize the spectral error within 2000 iterations. During the fitting process, the tissue parameters were nonlinearly constrained to make sure that the corresponding OPs did not exceed the ranges shown in Table 1.

In our previous preliminary study,^40^ the proposed method was validated to quantify OPs of a three-layered tissue phantom. Details are described in the Supplementary Material, and errors for extracted OPs of all layers were below 15%.

To assess the accuracy of the iterative curve fitting method using realistic head models, we generated 15 sets of test spectra using randomly picked tissue parameters and each of the nine subjects’ ANN model. We added to the test spectra 14 sets of random noise whose CV was similar to those estimated from *in-vivo* measurements to simulate the fluctuations of *in-vivo* spectra due to various sources including noise of the EMCCD readings, instability and variation of the probe-tissue coupling, and physiological changes of hemoglobin concentrations in the probed region. Therefore, there were 210 sets of test spectra with noise per subject, and 1890 sets of test spectra in total. Each set of the test spectra was fitted 20 times using different initial values as described above.

### 2.5 Multiple Solution

**Fig. 5:**
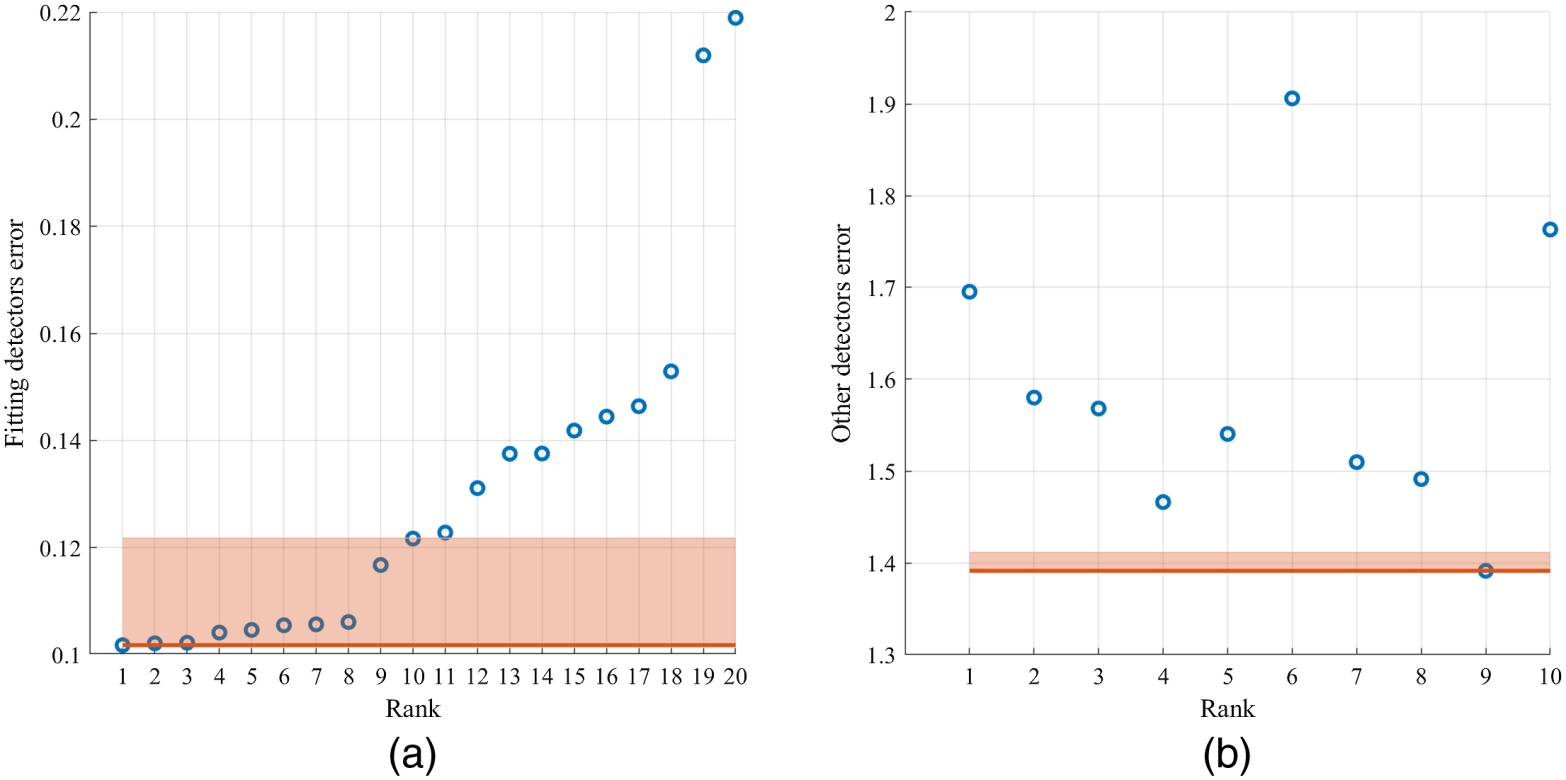
The process of choosing fitting solutions. (a) Spectral error of detectors included in the fitting, from which 10 fitting results were selected, and (b) spectral error of detectors not fitted in the 10 results selected in (a), from which only one result was selected. The shaded areas represent the range of 2% spectral error higher than the lowest error.

The fitting result with the smallest spectral error among the 20 times of fittings was typically chosen as the final result. However, since there was noise in the measured reflectance spectra, when multiple fitting results showed spectral errors to be within the noise level of each other, we could not verify which one was the best solution. The noise level of the NIRS system was estimated to be 2% by measuring static tissue phantoms presenting reflectance intensities similar to *in-vivo* forehead. When the difference between spectral errors of multiple fitting results was <2%, we considered those fitting results as potential multiple solutions. To reduce the number of multiple solutions and increase the chance of getting a unique solution of OPs, we employed spectra measured from detectors that were not used in the iterative curve fitting as tie breakers (see Table 9 for detector combinations chosen for fitting *in-vivo* spectra). An example of choosing the best solution is shown in Fig. 5. Assume only spectra of detectors one to four were used in the fitting. We compared spectral errors of the 20 fittings and chose the results whose spectral errors were within 2% of the smallest error. Then we compared the spectral error of detectors not included in the fitting, i.e., detectors 5 and 6, and again chose the results with errors within 2% of the lowest error. If two or more solutions were chosen, they were considered multiple solutions.

## 3 Results

### 3.1 Performance of the ANN Model

Using a larger detector fiber NA to run MC simulations increases the number of photons collected by the fiber and hence the simulation efficiency. By using the NA-conversion model described in Sec. 2.2, we could reduce the launched photon number by about 10 times while keeping the noise at a similar level as shown in the first and third rows in Table 5.

**Table 5:** Comparison of CVs of MC-simulated reflectance between NA = 1.0 with NA-conversion and NA = 0.12.

Photon number	NA of fiber	Conversion to low NA	SDS (cm)					
			0.8	1.5	2.12	3	3.35	4.5
$10^{9}$	1.0	Yes	0.42%	0.80%	1.42%	3.60%	4.15%	8.21%
$10^{9}$	0.12	No	0.87%	2.60%	2.51%	7.21%	8.42%	25.80%
$10^{10}$	0.12	No	0.45%	0.87%	0.86%	1.79%	2.64%	8.30%
$2\times 10^{11}$	0.12	No	0.10%	0.19%	0.19%	0.40%	0.59%	1.86%

The WMC simulations of 2808 $\mu_{s}^{\prime }$ combinations in the lookup table took about 24 h using seven NVIDIA GeForce RTX 2080. The generation of the 17.4 million training data from lookup table took 3 h. The training of ANN was done in 2 h. To assess the combined error introduced by the lookup table, NA-conversion model and trained ANN forward model, we simulated three spectra, each consisting of 35 wavelengths, for two subjects using MC simulations with $2\times 10^{11}$ photons and NA=0.12 detector fibers to serve as ground-truth reflectance values. The same OPs were input into the trained ANNs and resultant reflectance values were compared with the MC-simulated ground-truth values. The RMS spectral error for each detector SDS is listed in Table 6. We found that the error between the ANN output and the ground-truth MC simulation was comparable to the noise of MC simulations with about $10^{9}$ photons (see Table 5), while the speed of ANN was six orders of magnitude faster than MC simulations. For example, it took about 50 min to run MCX simulations with $10^{9}$ photons each to generate reflectance spectra consisting of 22 wavelengths, while using the ANN only took 2.6 ms.

**Table 6:** RMS percent error between MC-simulated and ANN-generated spectra average across two subjects and three spectra.

SDS (cm)	0.8	1.5	2.12	3	3.35	4.5
Error	2.00%	4.74%	5.02%	3.29%	3.03%	7.89%

Instead of training ANN forward models, the OP/reflectance data generated by MC simulations described in Sec. 2.2 could also be used to build an eight-dimensional lookup table. We tested using linear interpolation of the lookup table as the forward model, and found that spectral errors of the lookup table-based forward model were significantly larger than those of the ANN models for SDS equal to or longer than 3 cm, while the speed was forty times slower than ANN.

### 3.2 Test Spectra Fitting Results

**Fig. 6:**
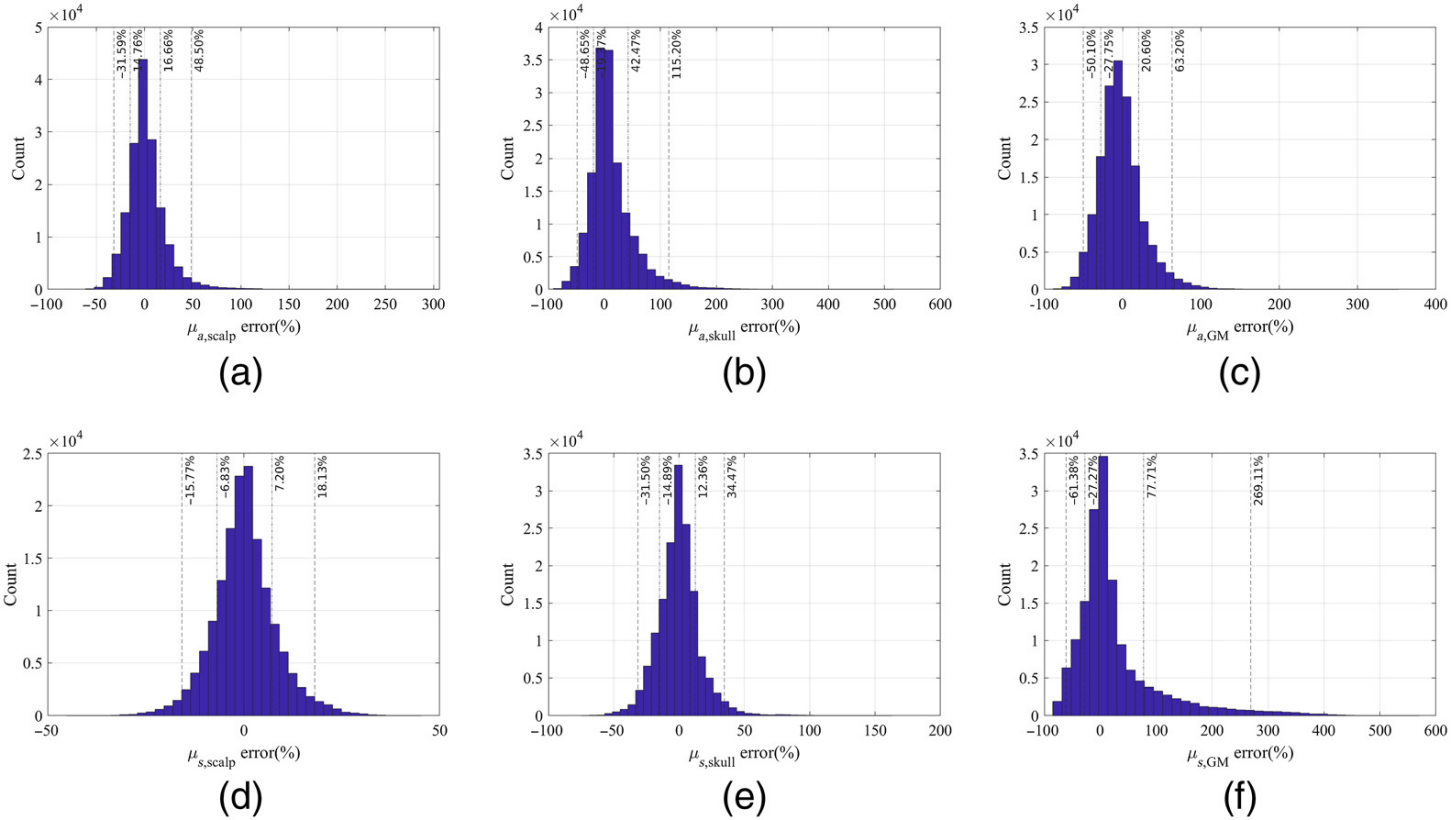
Histograms of relative errors in the six OPs quantified by fitting the test spectra of detectors 1 to 6 and choosing at most four multiple solutions. The dash-dotted lines and dash lines mark the 68% and 95% confidence intervals of the errors, respectively.

Fitting one set of test spectra with 20 different initial values was done in 4 min. Relative errors in each of the six OPs estimated from iterative curve fitting of 1890 test spectra, 22 wavelengths per spectrum, were compiled to form a histogram for one OP as shown in Fig. 6. Multiple solutions as defined in Sec. 2.5, if existed, were included as separate data points. The histogram of errors could be taken as an estimation of the probability density function of the fitting errors. We defined the range of errors that covered 68% or 95% of the fitting results as confidence intervals of each estimated OP. The errors in OPs corresponding to 68% confidence intervals for each detector combination and OP are listed in Table 7.

**Table 7:** Ranges of errors (%) in the fitted OPs corresponding to 68% confidence intervals.

Detector combinations	$\mu_{a,\text{scalp}}$	$\mu_{s,\text{scalp}}^{\prime }$	$\mu_{a,\text{skull}}$	$\mu_{s,\text{skull}}^{\prime }$	$\mu_{a,\mathrm{GM}}$	$\mu_{s,\mathrm{GM}}^{\prime }$
346	−19∼21	−9∼12	−27∼42	−15∼19	−30∼25	−28∼118
1234	−14∼20	−7∼7	−29∼41	−14∼18	−28∼25	−27∼111
2345	−17∼21	−9∼10	−26∼38	−14∼18	−29∼25	−28∼99
12345	−15∼20	−7∼8	−27∼40	−14∼17	−27∼23	−28∼89
123456	−15∼17	−7∼7	−19∼42	−15∼12	−28∼21	−27∼78

To compare errors between quantified OPs using subject-specific models and a general model, we used Colin 27 average brain model^41^ to train an ANN. The 1890 test spectra were fitted using the Colin 27 ANN and OPs’ errors were calculated. Table 8 shows a comparison of ranges of errors in the fitted OPs between each subject’s own ANN model and the Colin 27 ANN model. The ranges of errors are significantly larger for the Colin 27 model. We also fitted *in-vivo* spectra with the Colin 27 ANN model, and fitted OPs were different from those fitted with each subject’s own ANN even considering the OPs’ error ranges. The results suggest that the subject-specific model is needed for more accurate quantification of tissue OPs.

**Table 8:** Comparison between fitting test spectra with subject-specific models and the general Colin 27 model. Ranges of errors (%) in the fitted OPs correspond to 68% confidence intervals. Spectra of detectors 1 to 6 were used.

Fitting model	$\mu_{a,\text{scalp}}$	$\mu_{s,\text{scalp}}^{\prime }$	$\mu_{a,\text{skull}}$	$\mu_{s,\text{skull}}^{\prime }$	$\mu_{a,\mathrm{GM}}$	$\mu_{s,\mathrm{GM}}^{\prime }$
Each subject’s own model	−15∼17	−7∼7	−19∼42	−15∼12	−28∼21	−27∼78
Colin 27	−32∼92	−34∼13	−1∼162	−42∼70	−44∼57	−73∼42

### 3.3 *In-Vivo* Fitting Results

When fitting *in-vivo* spectra, we found that in some subjects not all detectors showed a small (<15%) spectral error. This might be caused by mismatches between actual human heads and the 3D tissue models built. The model assumes homogeneous OPs within each compartment, while in reality, there could be some inhomogeneities such as blood vessels under a detector or multiple detectors, making it hard to achieve small spectral errors for all detectors simultaneously. Table 9 shows spectral errors for each subject when spectra from different detector combinations were fitted. We chose the combination that included the most detectors and achieved a spectral error below 15% as the final fitting result. The chosen detector combination for each subject is marked in bold in Table 9, and the corresponding modeled and measured spectra are shown in Figs. S1–S5.

**Table 9:** Average spectral errors of each subject’s fitting results using different detector combinations. Only errors contributed by the fitting detectors are counted. The detector combination chosen for each subject is shown in bold.

Detector combination	Subject				
	1	2	3	4	5
346	4.9%	**5.1%**	5.1%	25.1%	22.0%
1234	7.1%	26.3%	**13.2%**	9.5%	**7.2%**
2345	11.9%	37.5%	14.8%	7.3%	18.4%
12345	11.9%	35.5%	19%	**10.2%**	17.9%
123456	**11.3%**	32.8%	19.3%	29.3%	29.2%

**Fig. 7:**
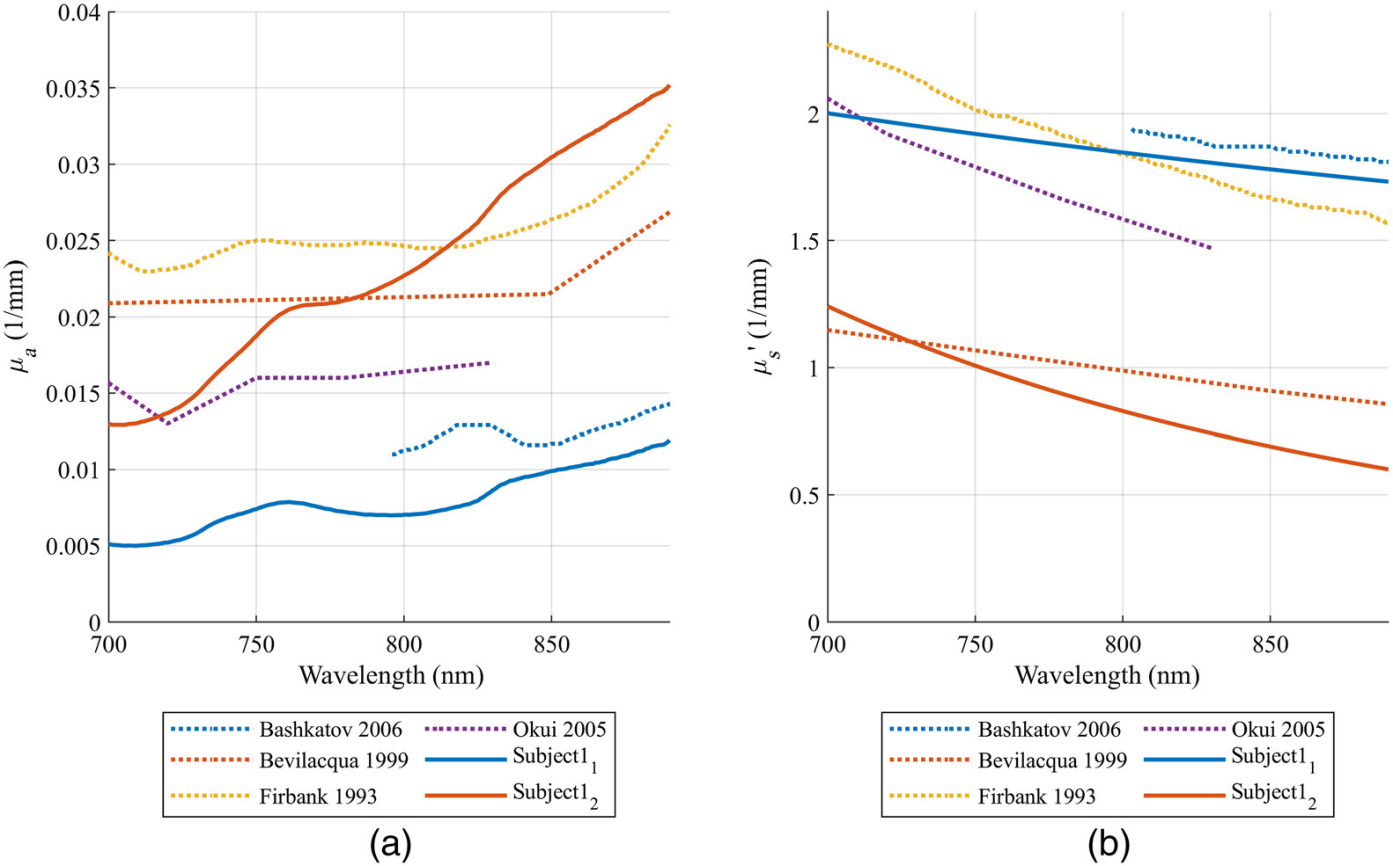
Comparison of the quantified skull (a) $\mu_{a}$ and (b) $\mu_{s}^{\prime }$ of subject 1 to the literature reported OPs.^34^^,^^42^^–^^44^

**Fig. 8:**
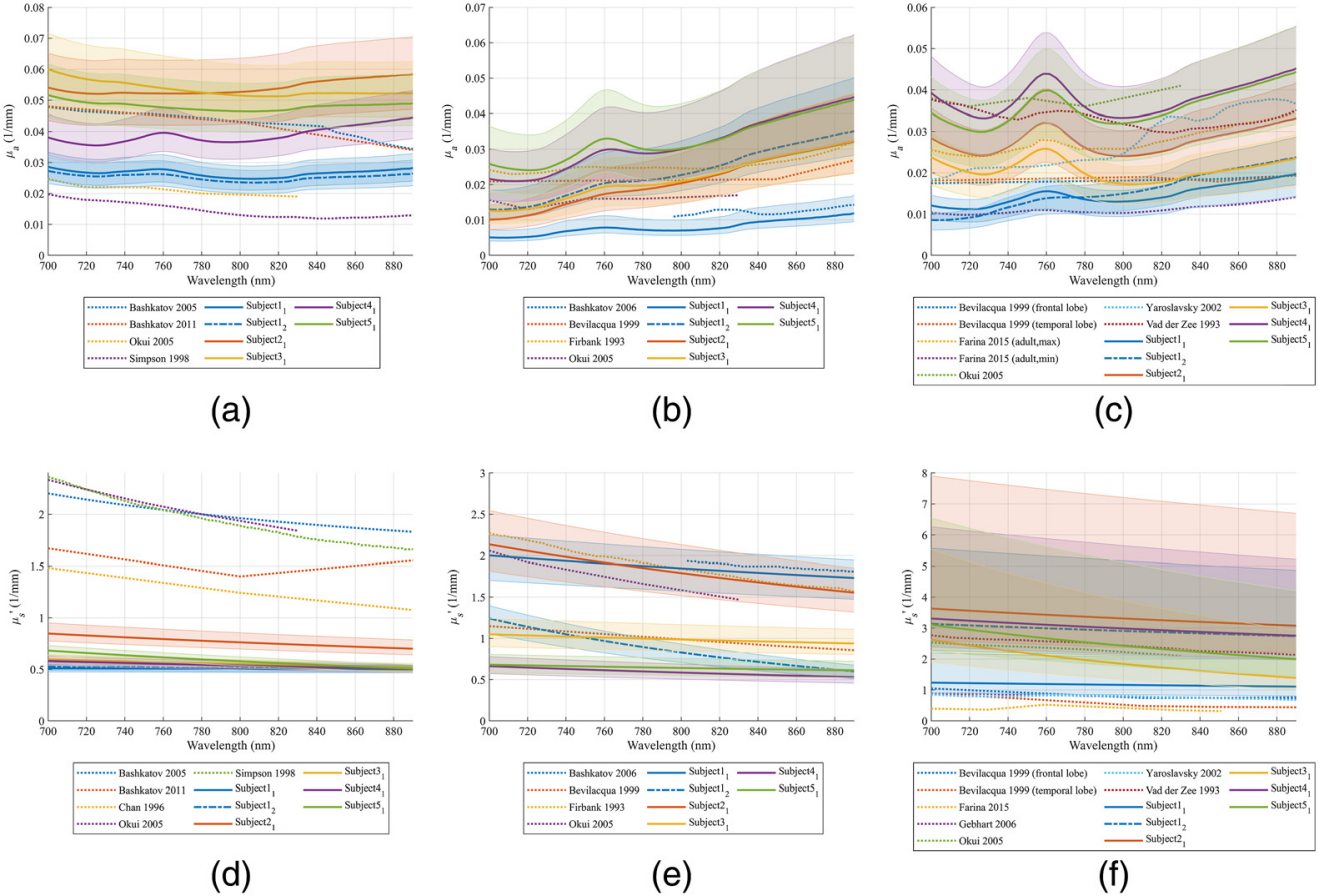
The fitted OPs and the literature reported value (a) $\mu_{a,\text{scalp}}$,^34^^,^^45^^–^^47^ (b) $\mu_{a,\text{skull}}$,^34^^,^^42^^–^^44^ (c) $\mu_{a,\mathrm{GM}}$,^17^^,^^34^^,^^43^^,^^48^^,^^49^ (d) $\mu_{s,\text{scalp}}^{\prime }$,^34^^,^^46^^,^^47^^,^^50^ (e) $\mu_{s,\text{skull}}^{\prime }$,^34^^,^^42^^–^^44^ and (f) $\mu_{s,\mathrm{GM}}^{\prime }$.^17^^,^^34^^,^^35^^,^^43^^,^^48^^,^^49^ The range with a 68% confidence level for each OP is shown in shades.

Fitting *in-vivo* spectra by the procedure described in Secs. 2.4 and 2.5 resulted in only one solution for all subjects except for subject 1. The two solutions of subject 1’s fitting results showed similar scalp OPs. In Fig. 7, we compare the skull OPs of the two solutions to literature-reported values. Solution 1 had a slightly smaller $\mu_{a,\text{skull}}$ compared with values reported in the literature, while solution 2 had a slightly smaller $\mu_{s,\text{skull}}^{\prime }$ compared with values reported in the literature. Since the $\mu_{s,\text{skull}}^{\prime }$ from both solutions were close to literature-reported values, we considered both of them as final results for subject 1. As a summary, OPs of the five subjects are compared with the literature-reported values in Fig. 8.

## 4 Discussion

### 4.1 Fitting Results

**Fig. 9:**
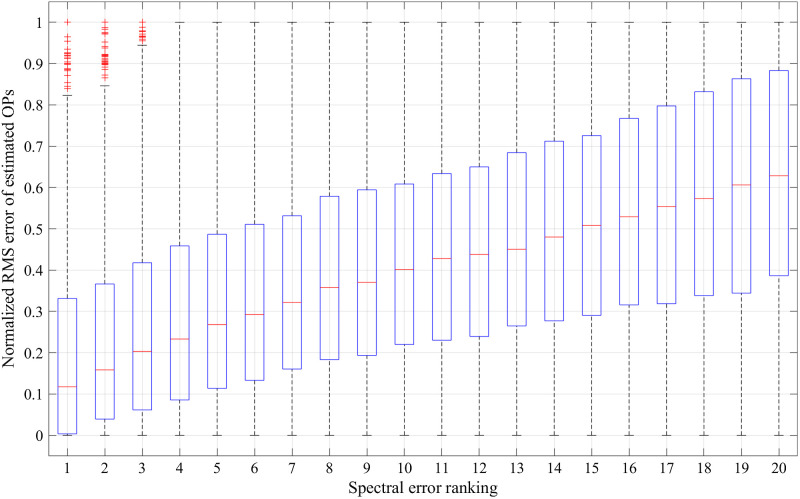
Boxplot of normalized OP errors to compare the 20 fitting results ranked according to spectral errors. The results shown were from fitting the 1890 test spectra sets using detectors 1 to 6, and similar results were obtained from choosing other detector combinations.

We validated the process of choosing the fitting result with the smallest spectral error out of the 20 fittings based on results of fitting the 1890 test spectra sets. For each test spectra set the 20 fitting results were ranked according to the spectral error. To compare errors of estimated OPs between the 20 fitting results we calculated an RMS percentage error of OPs estimated by each fitting over the 22 fitted wavelengths and six OPs, and normalized the RMS errors of the 20 fitting results to the range between 0 and 1. Normalizing the OP errors enabled combining results of the 1890 test spectra sets since errors in OPs varied substantially across the test data. Afterward, the normalized OP errors of the fitting result with the same spectral error ranking from each of the 1890 test spectra sets were compiled together. Figure 9 shows a boxplot of normalized OP errors from results of all the 1890 test spectra sets to compare the 20 fitting results ranked according to spectral errors. It can be seen that the normalized OP errors are smaller in fitting results with smaller spectral errors. The result suggests that choosing the fitting result with the smallest spectral error leads to overall smaller errors in the six estimated OPs.

**Fig. 10:**
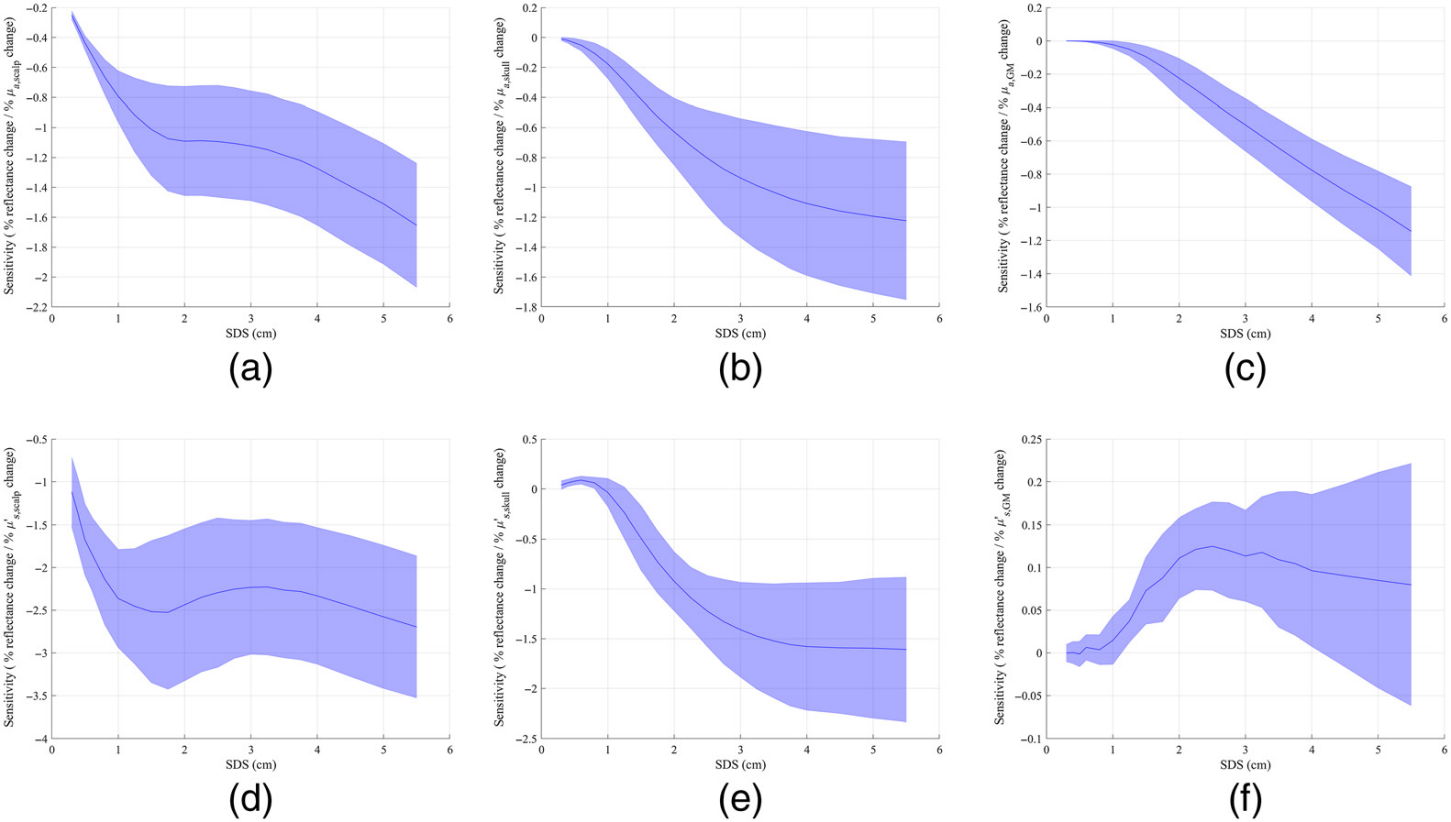
Average sensitivity of detectors at various SDSs to (a) $\mu_{a,\text{scalp}}$, (b) $\mu_{a,\text{skull}}$, (c) $\mu_{a,\mathrm{GM}}$, (d) $\mu_{s,\text{scalp}}^{\prime }$, (e) $\mu_{s,\text{skull}}^{\prime }$, and (f) $\mu_{s,\mathrm{GM}}^{\prime }$. Results were obtained from simulations using nine subjects’ head models.

To investigate sources of errors in quantified OPs, we also fitted the same 135 sets of simulated test spectra without added noise. Ranges of errors in OPs (shown in Table S2) were not significantly lower than those with added spectral noise (Table 7). Since the test spectra were generated using the same ANN as used in the iterative curve fitting, ideally a perfect fit should be achievable. However, the average spectral error of test spectra fitting results was 1.78% rather than 0%. This is probably caused by noise of MC simulations, which the trained ANN models also inherit. During iterative curve fitting the optimization algorithm needs to calculate partial derivatives of the reflectance with respect to individual OPs to find the global minimum. As shown in Fig. 10, the sensitivity of CW NIRS measurements to deep tissue OPs is very low, which means that the reflectance hardly changes given a small perturbation in one OP. When the noise in MC-simulated or ANN output reflectance is comparable to or even larger than the reflectance changes due to the small perturbation in an OP, the ability of iterative curve fitting to find the global minimum could be hindered. See Fig. S6 for an example of trends in the ANN output reflectance at the six SDSs when only one of the six OPs varies and the other OPs remain constant. It can be seen that the cases with low sensitivity (all SDSs in the case of $\mu_{s,\mathrm{GM}}^{\prime }$ and short SDSs in cases of $\mu_{a,\mathrm{GM}}$ and $\mu_{s,\text{skull}}^{\prime }$) show rugged or not monotonic trends in the reflectance with varying OP values. If the sensitivity of measured NIRS data to deep tissue OPs could be improved, e.g., using TR or FD NIRS systems, the influence of MC noise and ANN output errors could be reduced, and the errors of the fitted OPs may be improved.

The comparison of the fitted OPs and the literature reported values is shown in Fig. 8. All OPs except for $\mu_{s,\text{scalp}}^{\prime }$ are within the ranges reported in the literature. Although $\mu_{s,\text{scalp}}^{\prime }$ is much lower than the literature reported values, $\mu_{a,\text{scalp}}$ is not overestimated. Therefore, the underestimation of $\mu_{s,\text{scalp}}^{\prime }$ is not due to cross-talk between $\mu_{a,\text{scalp}}$ and $\mu_{s,\text{scalp}}^{\prime }$. Considering that the SDSs used in this study ranged from 0.8 to 4.5 cm and the spatial resolution of the 3D model was relatively low with a 0.93-mm edge length, the scalp was not segmented into finer layers such as epidermis, dermis, and subcutaneous tissue. The scattering phase functions used in MC simulations were set to the conventional Henyey–Greenstein phase function with a constant anisotropy factor. Quantification of $\mu_{s,\text{scalp}}^{\prime }$ could be improved by detecting spectra at shorter SDS and using more realistic structural and optical parameters for skin.

It is worth mentioning that the quantified $\mu_{a,\text{scalp}}$, $\mu_{a,\text{skull}}$, $\mu_{s,\text{skull}}^{\prime }$, and $\mu_{a,\mathrm{GM}}$ show high variabilities among subjects even considering the uncertainties shown in Fig. 8. The confidence interval for $\mu_{s,\mathrm{GM}}^{\prime }$ is too large, which prevents reliable quantification of $\mu_{s,\mathrm{GM}}^{\prime }$. The high intersubject variability underpins the importance of quantifying the tissue OPs of each subject to more accurately calculate partial pathlengths for fNIRS applications or determine the fraction of photon energy delivered to the brain for therapeutic applications.

### 4.2 Feasibility

The process of building a 3D head model is done manually and costs no more than 1 h. Simulations of 2808 $\mu_{s}^{\prime }$ combinations in the lookup table for generating ANN training data cost about 24 h using 7 NVIDIA GeForce RTX 2080. The measurement of NIRS spectra can be done in 1 h and can be done at the same time while the simulation is running. The generation of training data from the lookup table can be done in 3 h. The training of ANN can be done in 2 h. The fitting for one target spectra can be done in 4 min. The whole quantifying process can be done within 2 days after taking the MRI scan.

### 4.3 Future Work

We use iterative curve fitting in this study, whose performance is affected by the smoothness of the ANN output. Random noise in MC simulation results could be reduced using filtering techniques.^48^ Using other optimization algorithms, e.g., genetic algorithm, which do not rely on the gradient of the loss function might help reduce the number of multiple solutions.

The uncertainties of $\mu_{a,\mathrm{GM}}$ and $\mu_{s,\mathrm{GM}}^{\prime }$ are relatively large compared with the other OPs. To improve the sensitivity of the GM OPs, one may consider using TR or FD NIRS systems, as in Refs. 16–18. With the help of each subject’s own model and the precise MC simulations, the accuracy of quantified OPs could be improved.

The edge length of one voxel in our voxel-based model is 0.93 mm, which is relatively large compared with the 0.8 cm SDS. Tran et al.^51^ proposed a pipeline for generating mesh-based models from MRI images with smooth surfaces for each layer. With a mesh-based MC simulation tool, they showed that the travel distance of the photons in GM is overestimated compared with the voxel-based model. This may affect the simulated reflectance spectra. Switching to the mesh-based model may help improve the match between the real head and the model.

## 5 Conclusion

This study aimed to use a multidistance CW NIRS system to quantify the OPs of the scalp, skull and GM in the human head *in-vivo*. MC simulations were performed to generate reflectance spectra given OPs and a voxel-based tissue model of each subject’s head to improve the consistency between the subject and the model. Lookup tables and white MC method were employed to efficiently generate numerous data for training ANNs that replaced MC simulations in iterative curve fitting of measured reflectance spectra. The average error between ANN outputs and corresponding MC simulations was under 10%. Iterative curve fitting was used to optimize the solution. The curve fitting could be done in several minutes with the help of the ANNs. We performed fittings on thousands of simulated test spectra to estimate the confidence interval of each fitted OP. The OPs of the scalp, skull, and gray matter at the right forehead were quantified for five subjects. The scalp $\mu_{s}^{\prime }$ was underestimated while the other OPs were within the ranges reported in the literature. Differences in estimated OPs between different subjects are larger than the confidence intervals, which suggests the importance of quantifying the OPs for each subject.
