# Supplementary material for: Quantifying tissue optical properties of human heads in vivo using continuous-wave near-infrared spectroscopy and subject-specific three-dimensional Monte Carlo models
Source: J Biomed Opt. 2022 Jun 22;27(8):083021. doi: 10.1117/1.JBO.27.8.083021 (PMC9214577; doi:10.1117/1.JBO.27.8.083021)
Supplement: Supplementary file 1 [file JBO_027_083021_SD001.pdf]

# Supplementary Material

1. Validation of the proposed method on multi-layered tissue mimicking phantoms

We experimentally validated the proposed method to quantify OPs of multi-layered tissue phantom. A three-layered slab phantom was constructed and measured by the CW NIRS system, calibrated with the same homogeneous phantoms and procedure described in Sec. 2.4, and the OPs of the layers were quantified by iterative curve fitting. The three-layered phantom was made of PDMS, TiO2 and India ink. The forward model of the iterative curve fitting was Monte Carlo simulation. The $\mu_a$ of each layer of the phantom was modeled by the ink concentration, while the $\mu_s$ was modeled by A and K, thus there were 9 parameters to be adjusted during iterative curve fitting. The errors of quantified OPs are shown in Table S1, with all errors below 15%. This demonstrates the accuracy of the CW NIRS system and the data process pipeline using a simple multi-layered slab model.

**Table S1** Errors of quantified OPs of a three-layered phantom.

| Phantom layer | Layer 1 | Layer 2 | Layer 3 |
|---|---|---|---|
| $\mu_a$ error | 10.98% | 3.95% | 14.20% |
| $\mu_s$ error | 10.99% | 8.63% | 7.94% |
| Thickness (mm) | 3.0 | 7.0 | >50.0 |

2. Measured and best-fit *in-vivo* spectra of five healthy subjects

Five healthy subjects' *in-vivo* measured spectra and simulated spectra with fitted OPs are shown in Fig. S1-S5. The subjects' spectra were fitted with different detector combinations to make the average spectral error of chosen detectors below 15%.

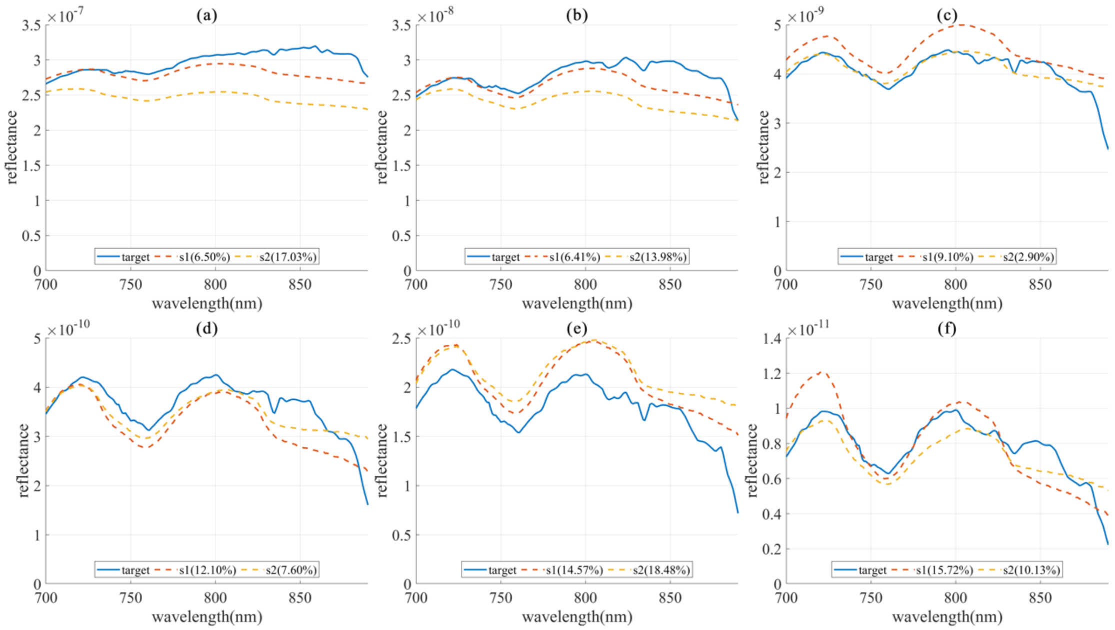


**Figure S1** *In-vivo* fitting results (s1: solution 1, s2: solution 2) for subject 1 fitted with detectors 123456: (a) SDS=0.8 cm, (b) SDS=1.5 cm, (c) SDS=2.12 cm, (d) SDS=3 cm, (e) SDS=3.35 cm, and (f) SDS=4.5 cm.

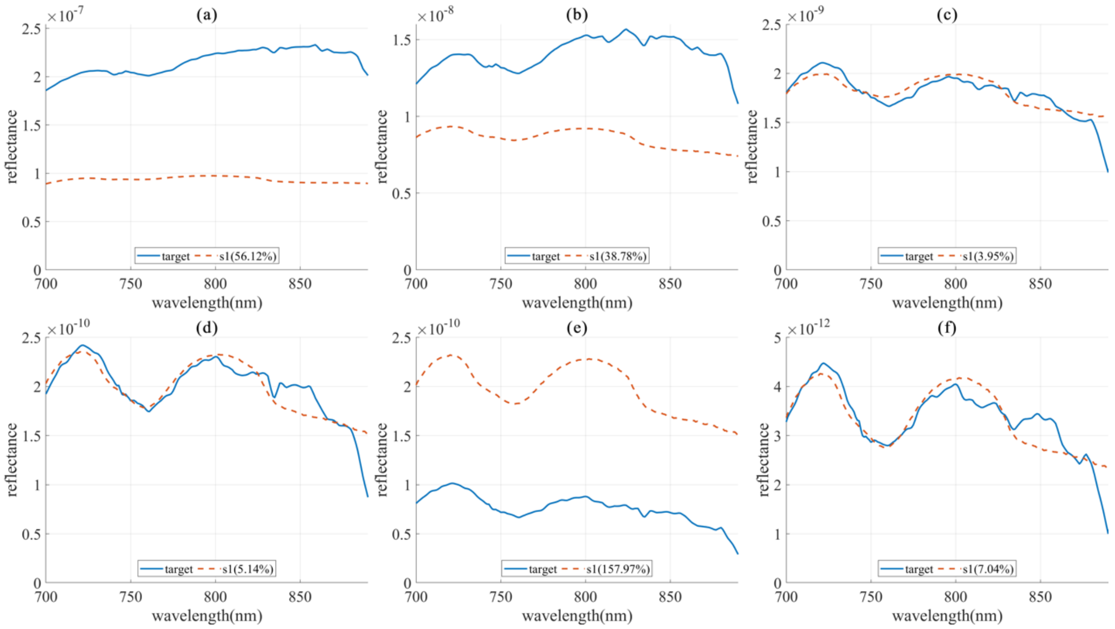


**Figure S2** *In-vivo* fitting results for subject 2 fitted with detectors 346: (a) SDS=0.8 cm, (b) SDS=1.5 cm, (c) SDS=2.12 cm, (d) SDS=3 cm, (e) SDS=3.35 cm, and (f) SDS=4.5 cm.

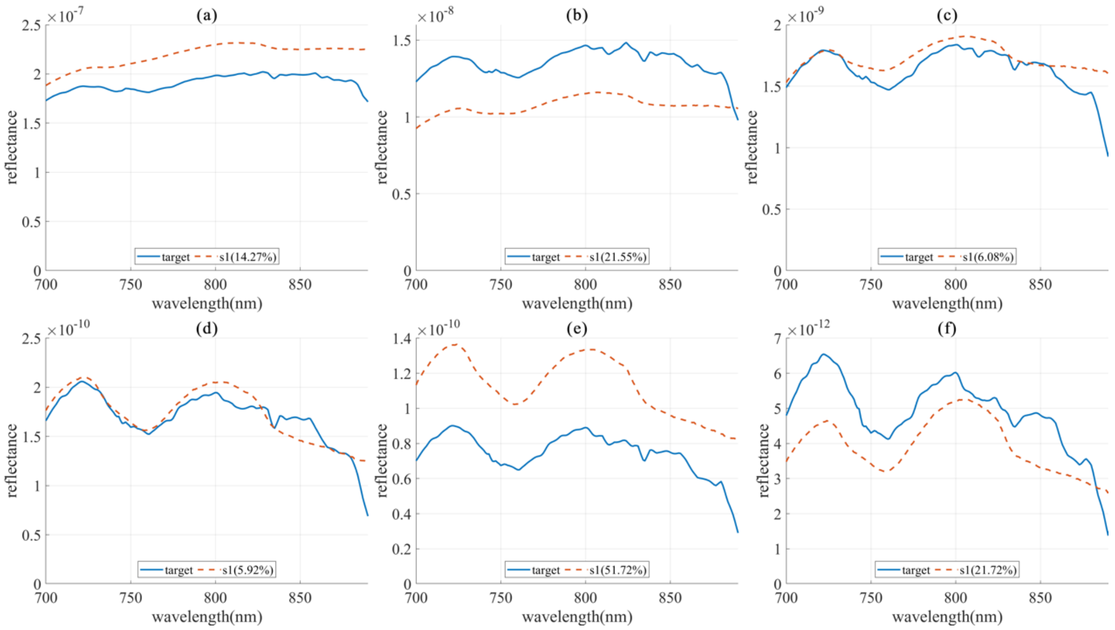


**Figure S3** *In-vivo* fitting results for subject 3 fitted with detectors 1234: (a) SDS=0.8 cm, (b) SDS=1.5 cm, (c) SDS=2.12 cm, (d) SDS=3 cm, (e) SDS=3.35 cm, and (f) SDS=4.5 cm.

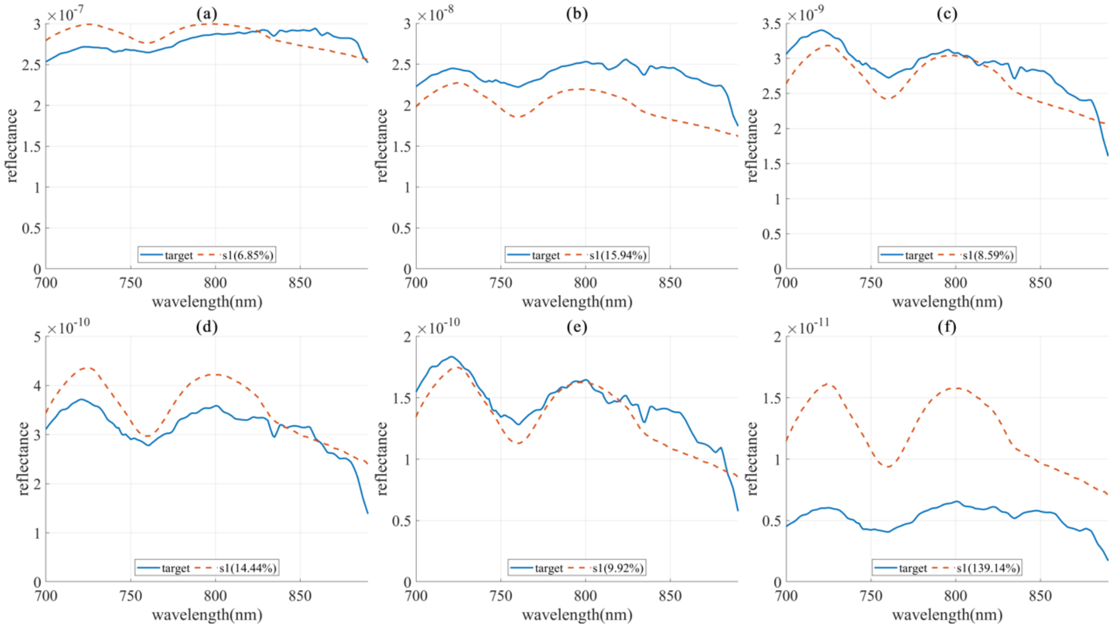


**Figure S4** *In-vivo* fitting results for subject 4 fitted with detectors 12345: (a) SDS=0.8 cm, (b) SDS=1.5 cm, (c) SDS=2.12 cm, (d) SDS=3 cm, (e) SDS=3.35 cm, and (f) SDS=4.5 cm.

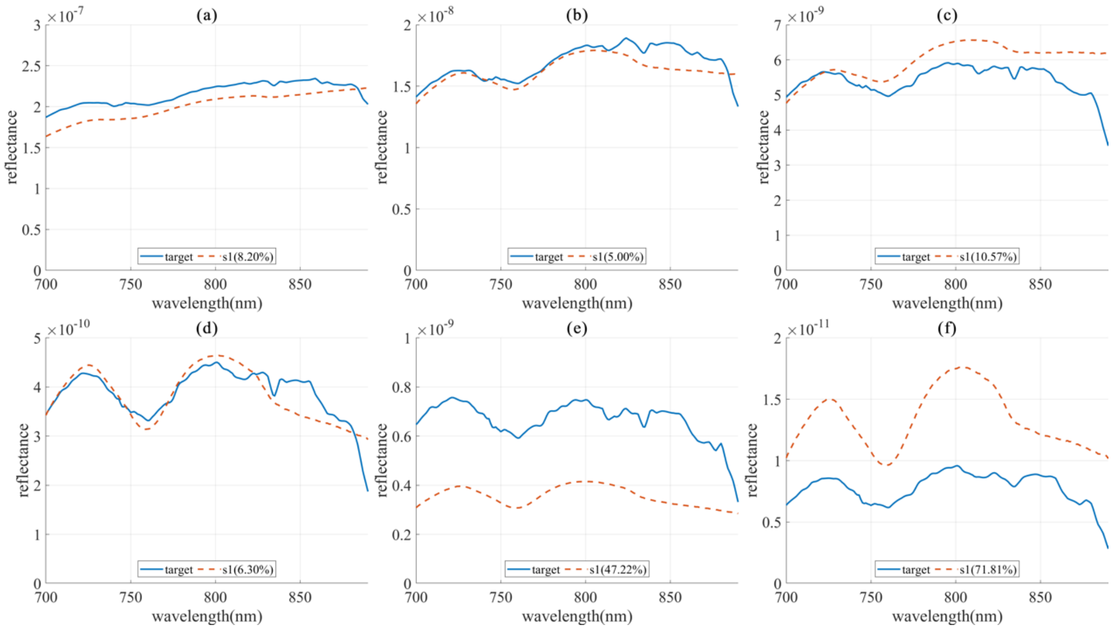


**Figure S5** *In-vivo* fitting results for subject 5 fitted with detectors 1234: (a) SDS=0.8 cm, (b) SDS=1.5 cm, (c) SDS=2.12 cm, (d) SDS=3 cm, (e) SDS=3.35 cm, and (f) SDS=4.5 cm.

## 3. Errors in quantified OPs from fitting the 135 sets of simulated test spectra without added noise

For each of the 9 subjects, 15 sets simulated spectra without additional errors were iteratively curve fitted. The relative errors for each OP were calculated at each of 22 wavelength points per set of spectra and merged from the 135 sets of fitted spectra. The errors in fitted OPs corresponding to 68% confidence intervals for each detector combination are listed in Table S2. Compared to the OP errors for simulated spectra with noise as shown in Table 7, the OP errors for spectra without noise are smaller especially for $\mu_{a,GM}$ and $\mu_{s,GM}$. This difference could be attributed to additional spectral noises due to the system noise and physiological changes of hemoglobin concentrations in the probed region.

**Table S2** Ranges of errors (in %) in the fitted OPs for target spectra without noise under 68% confidence interval.

| Detector combinations | $\mu_{a,scalp}$ | $\mu'_{s,scalp}$ | $\mu_{a,skull}$ | $\mu'_{s,skull}$ | $\mu_{a,GM}$ | $\mu'_{s,GM}$ |
|---|---|---|---|---|---|---|
| 346 | -13~11 | -5~8 | -24~38 | -13~16 | -23~16 | -19~123 |
| 1234 | -8~10 | -4~4 | -21~31 | -9~12 | -22~13 | -16~78 |
| 2345 | -10~10 | -5~6 | -16~32 | -12~12 | -21~10 | -17~76 |
| 12345 | -7~9 | -3~3 | -17~23 | -8~11 | -19~11 | -17~50 |
| 123456 | -9~9 | -4~4 | -14~33 | -11~9 | -20~10 | -15~55 |

## 4. Trends in the reflectance at six SDSs when only one of the six OPs is varied

To illustrate the sensitivity of NIR reflectance spectra to each of the six OPs considered in this study, reflectance values of subject 1 were obtained by the ANN models and normalized while only one of the six OPs was varied (Fig. S6). The trends for $\mu_{a,scalp}$, $\mu_{s,scalp}$, $\mu_{a,skull}$ and $\mu_{s,skull}$ are rather smooth and monotonic, while for $\mu_{a,GM}$ with SDS shorter than 3 cm and $\mu_{s,GM}$, the trends are rugged and not monotonic.

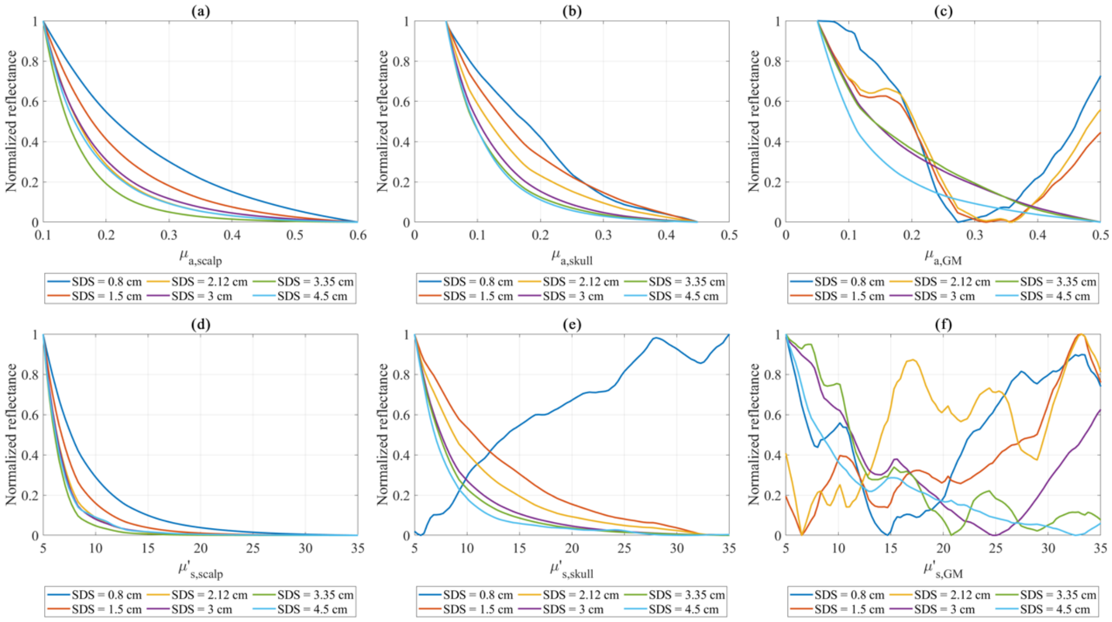


**Figure S6** Normalized reflectance from subject 1's ANN output when only (a) $\mu_{a,scalp}$, (b) $\mu_{a,skull}$, (c) $\mu_{a,GM}$, (d) $\mu'_{s,scalp}$, (e) $\mu'_{s,skull}$, and (f) $\mu'_{s,GM}$ was varied. The other OPs were set to each one's corresponding average value. The reflectance values were scaled to the range [0, 1] to aid the viewing since they span several orders of magnitude across the six SDSs.
